# Combined use of EMLA cream and Buzzy for peripheral intravenous cannulation in children: a randomized controlled trial

**DOI:** 10.3389/fped.2026.1816908

**Published:** 2026-07-23

**Authors:** Marco Persoglia, Irene Lapucci, Egidio Barbi, Manuela Giangreco, Paola Cogo, Giada Vittori, Daniela Slama, Anna Monsutti, Alice Martin, Giorgio Cozzi

**Affiliations:** 1 Department of Medicine, Surgery and Health Sciences, University of Trieste, Trieste, Italy; 2 Institute for Maternal and Child Health, IRCCS Burlo Garofolo, Trieste, Italy; 3 Department of Pediatrics, University Hospital of Udine, Udine, Italy

**Keywords:** catheterization peripheral, child, emotional stress, lidocaine, pain management, prilocaine, vibration

## Abstract

**Aim:**

Preventing pain and distress during peripheral intravenous cannulation in children is essential to avoid long-term anxiety or needle phobia. This study evaluated whether combining EMLA cream with the Buzzy device is more effective than EMLA alone in reducing procedural distress and pain.

**Materials and methods:**

We conducted a randomized controlled trial in two tertiary children's hospitals in Italy (March-October 2023). Children aged 4–12 years requiring intravenous cannulation were randomized to receive either EMLA alone or EMLA plus Buzzy. The primary outcome was distress, assessed by parents using the Children's Emotional Manifestation Scale (CEMS). Secondary outcomes included pain measured with the Faces Pain Scale-Revised (FPS-R) by children, parents, and healthcare operators, operator-rated distress, first-attempt success, and adverse events. Analyses followed an intention-to-treat basis.

**Results:**

Ninety-four children (median age 8 years) were enrolled: 47 in the EMLA plus Buzzy group and 47 in the EMLA alone group. Parent-rated distress was significantly lower with EMLA plus Buzzy (median CEMS 6, IQR 5–7) than with EMLA alone (9, IQR 7–12; *p* = 0.001). Pain scores and first-attempt success rates were similar. No severe adverse events occurred.

**Conclusion:**

Combining EMLA and Buzzy significantly reduced procedural distress perceived by parents without affecting pain scores.

## Introduction

Venipuncture and peripheral intravenous cannulation are among the most common painful procedures in hospitalized children (1). In particular, peripheral venous catheter placement is both painful and associated with significant distress in children (2). Moreover, if procedural pain is not managed, children may later develop anxiety, needle phobia, or an increased perception of pain during future procedures. This occurs because negative procedural experiences can generate an emotional memory of pain and reinforce the cycle of fear–pain–distress over time (3). In this regard, international guidelines emphasize the importance of adopting strategies to minimize both pain and distress during needle procedures (4, 5).

Therefore, a range of non-pharmacological and pharmacological strategies has been developed to reduce pain and distress associated with painful procedures (4). EMLA cream (lidocaine 2.5%, prilocaine 2.5%) is one of the most widely used topical anesthetics in Europe for peripheral intravenous cannulation (6, 7). It is effective in blocking nociceptive input and has been shown to be safe and effective in pediatric intravenous cannulation procedures, requiring at least 60 min of occlusive application and being well tolerated. Among the available topical anesthetics, EMLA cream has the highest number of studies demonstrating its efficacy for peripheral intravenous cannulation in children (6–8). However, this analgesic strategy does not target the distress component associated with these procedures.The emotional component, mainly anxiety and fear, often makes needle procedures more unpleasant for children than the nociceptive pain itself. For this reason, managing pain alone is not sufficient; addressing the emotional component with non-pharmacological strategies is essential to reduce distress and improve the child's overall procedural experience (4).

The Buzzy (MMJ Labs, Atlanta, GA, USA) device is a small (8 × 5 × 2.5 cm) reusable plastic device shaped like a bee or a ladybug that incorporates ice-pack wings and a battery-powered vibration motor. It combines distraction, cold, and vibration to provide analgesia during painful procedures (8–10). Its effect is generally explained by sensory modulation mechanisms consistent with the gate control theory of pain, according to which competing non-nociceptive sensory input may attenuate nociceptive signal processing at the spinal level (11, 12). In addition to its peripheral effects, the combined sensory stimulation may help shift the child's attention away from the procedure and reduce anticipatory anxiety. A practical advantage of Buzzy is that, unlike topical anesthetics such as EMLA cream, it can be applied immediately before cannulation.

Over recent years, Buzzy has been studied as a non-pharmacological approach to reduce pain and distress during common pediatric procedures, including venipuncture, peripheral intravenous cannulation, and intramuscular injections (10, 13–16). Available evidence also suggests a potential benefit in children with cognitive disabilities, a group known to be particularly susceptible to procedural distress (9). Most available studies have evaluated Buzzy in comparison with other non-pharmacological techniques or to procedures performed without any analgesic intervention (10, 14, 15). To the best of current knowledge, no studies have evaluated whether the combined use of EMLA cream and Buzzy reduces procedural distress and pain for peripheral intravenous cannulation in children. We hypothesised that the combined use of EMLA cream and Buzzy could help decrease distress in children and offer a synergistic effect on pain reduction.

Therefore, this study aimed to assess, in children undergoing peripheral intravenous cannulation, whether the addition of Buzzy to topical anesthesia (EMLA cream), compared with EMLA alone, reduces procedural distress and pain.

## Materials and methods

This was a multicenter, open-label, parallel-arm, randomized controlled clinical trial. The open-label design was adopted because blinding was not feasible due to the visible and physical nature of the Buzzy device. The multicenter setting was chosen to enhance the generalizability of the results and to facilitate patients' recruitment across different clinical settings. It was conducted from March 1st to October 2nd, 2023, at two tertiary-level university-affiliated pediatric hospitals in Italy. The study was approved by the Regional Ethics Committee (CEUR-2020-Sper-067) and conducted in accordance with the Declaration of Helsinki. The trial was prospectively registered on ClinicalTrials.gov (NCT04695054; https://clinicaltrials.gov/study/NCT04695054) in January 2021. The full study protocol is available on ClinicalTrials.gov. Patient recruitment was conducted between March 2023 and October 2023. The interval between registration and study completion reflects the time required for protocol finalization, ethical approval, staff training, and coordination between participating centers. All parents of the enrolled children provided written informed consent; a dedicated information document was also provided for children over 6 years of age.

Inclusion criteria:

- Age between 4 and 12 years
- Undergoing peripheral intravenous cannulation
- Application of EMLA cream at least 60 min before the procedure

Exclusion criteria

- Cognitive impairment
- Presence of skin lesions preventing correct positioning of the Buzzy device
- Cold hypersensitivity disorders (e.g., sickle cell disease, Raynaud's phenomenon)
- Use of analgesic medications within 8 h prior to the procedure.

Subjects were randomly assigned to one of two study arms:

- EMLA plus Buzzy group, in which the Buzzy device (MMJ Labs, Atlanta, GA, USA) was applied approximately 5 cm above the cannula insertion site, at least 30 s before insertion, and kept active until the procedure was completed. The device was used with the ice-pack wings positioned on its back. EMLA cream was applied at least 60 min before, according to standard clinical practice.
- EMLA alone group, in which only EMLA was applied, 60 min before the procedure.

Randomization was performed using a computer-generated sequence with a 1:1 allocation ratio, in blocks of 10 and stratified by enrolment site. Allocation was managed using opaque, sequentially numbered sealed envelopes prepared by a researcher not involved in patient enrolment. After verifying the eligibility criteria and obtaining informed consent, the envelope with the lowest available number was opened to determine the subject's assignment to the experimental group or the control group. Due to the nature of the intervention, blinding of patients and healthcare operators was not feasible.

All procedures were performed by trained pediatric nurses. Nurses used verbal comforting speech during the procedures according to the child's age, and parents were always involved in comforting and supporting their children. The procedures were performed with the child seated on a chair or on the parent's lap, avoiding a lying position, which is well known to increase pain and distress in children during needle procedures (4). The use of external distraction devices by either staff or parents was not permitted; in particular, electronic devices (computer, tablet, smartphone, television), soap bubbles, books, toys, or stuffed animals were not allowed.

### Outcomes

The primary outcome of the study was to compare the level of procedural distress in the two groups, assessed by parents immediately after the procedure using the CEMS (Children's Emotional Manifestation Scale).

Secondary outcomes included:.

- Child self-reported pain using the FPS-R (Faces Pain Scale Revised).
- Procedural distress using the CEMS scale completed by the healthcare operator
- Pain rated by parents and healthcare operators (FPS-R).
- Procedural success at first attempt.
- Number and type of adverse events.

The Children's Emotional Manifestation Scale (CEMS) is an observational scale used to evaluate children's behavioral and emotional reactions during painful or stressful procedures. It includes five domains (facial expression, vocalization, motor activity, interaction and cooperation), each scored from 1 to 5. The total score ranges from 5 to 25, with higher scores indicating greater distress. The scale has been validated in children aged 7–12 years, showing good inter-rater reliability and internal consistency (17). Although it has not been formally validated in younger children, we considered it appropriate for use in this study because it is based on direct observation of behavioral manifestations rather than self-report.

The FPS-R scale consists of 6 faces, each aligned with expressions of increasing pain intensity at equal intervals, from which children select the one that best matches their pain. This information was then converted into a numerical value, ranging from 0 (no pain) to 10 (maximum pain) (18). The scale has been shown to be reliable in children, particularly in those aged 4–12 years (19).

Children aged 4–12 years were included, as they are typically able to use the FPS-R reliably and display observable behavioral responses during procedures. The upper age limit was chosen to restrict the sample to pre-adolescent children and to limit variability related to developmental changes during adolescence.

### Study conduct

Upon arrival at the Pediatric Day Hospital or Pediatric Clinic, the study was presented and explained to eligible patients and their caregivers by a physician researcher. Children meeting the inclusion and exclusion criteria were consecutively enrolled after written informed consent was obtained from the parents. Patient demographic and clinical data were recorded at baseline. Before the procedure, parents were briefly instructed in the use of the CEMS scale, and both children and parents were instructed in the use of the FPS-R scale. After confirming eligibility and obtaining informed consent, participants were assigned to the study groups by opening the next available sealed envelope. All procedures were performed by an experienced pediatric nurse. This included the application of EMLA 60 min before the procedure, the positioning and activation of the Buzzy device (when allocated), and the insertion of a 24-gauge peripheral venous catheter. After the procedure, outcome data were collected by the physician researcher. CEMS scores were obtained from parents and healthcare professionals, and FPS-R scores from children, parents, and healthcare professionals.

### Sample size calculation

The baseline hypothesis was that there would be a clinically significant difference of 3 points in the CEMS scale between the two groups, with estimated mean scores of 12 (SD = 5) in the EMLA plus Buzzy group and 15 (SD = 5) in the EMLA alone group. With a significance level (α) of 0.05, a power of 80% (β = 0.20), and an estimated effect size of 0.6, the required sample size was 94 participants (47 per group), as calculated using G*Power for the Wilcoxon-Mann–Whitney test.

### Statistical analysis

Statistical analysis was performed by intention-to-treat, including all randomized participants in the groups to which they were originally assigned, regardless of protocol adherence. Categorical variables are described in terms of absolute frequency and percentage. In contrast, for the statistical description of continuous variables, the median and interquartile range (i.e., the 25th and 75th percentiles) are used. To verify the success of randomization, a comparison was made between the two groups for the main characteristics of the enrolled sample. Gaussian distribution of the continuous variables was assessed using the Shapiro–Wilk test. To account for potential age-related differences in pain and distress responses, participants were stratified into two age groups (≤8 years and >8 years), and analyses were performed accordingly. Associations between categorical variables were evaluated by the Chi-square test or exact Fisher test, as appropriate. To compare the distribution of a continuous variable between the categories of a dichotomous variable, the non-parametric Wilcoxon-Mann–Whitney test was applied. A *p*-value < 0.05 was considered statistically significant. All statistical analyses were conducted using the SAS 9.4 software (SAS Institute Inc., Cary, NC, USA). No missing data were observed; therefore, no imputation was performed.

## Results

We assessed 104 patients for eligibility for the study. Two were excluded (1.9%): one for taking pain medication within 8 h before the procedure and one for cognitive impairment. Eight patients (7.7%) refused to participate. Ninety-four children were then enrolled, of whom 47 were randomized to the EMLA plus Buzzy group and 47 to the EMLA alone group. One child who was randomized to the EMLA alone group inadvertently received the Buzzy device; therefore, in accordance with the intention-to-treat principle, this participant was analyzed in the group to which they had originally been randomized.

**Figure 1:**
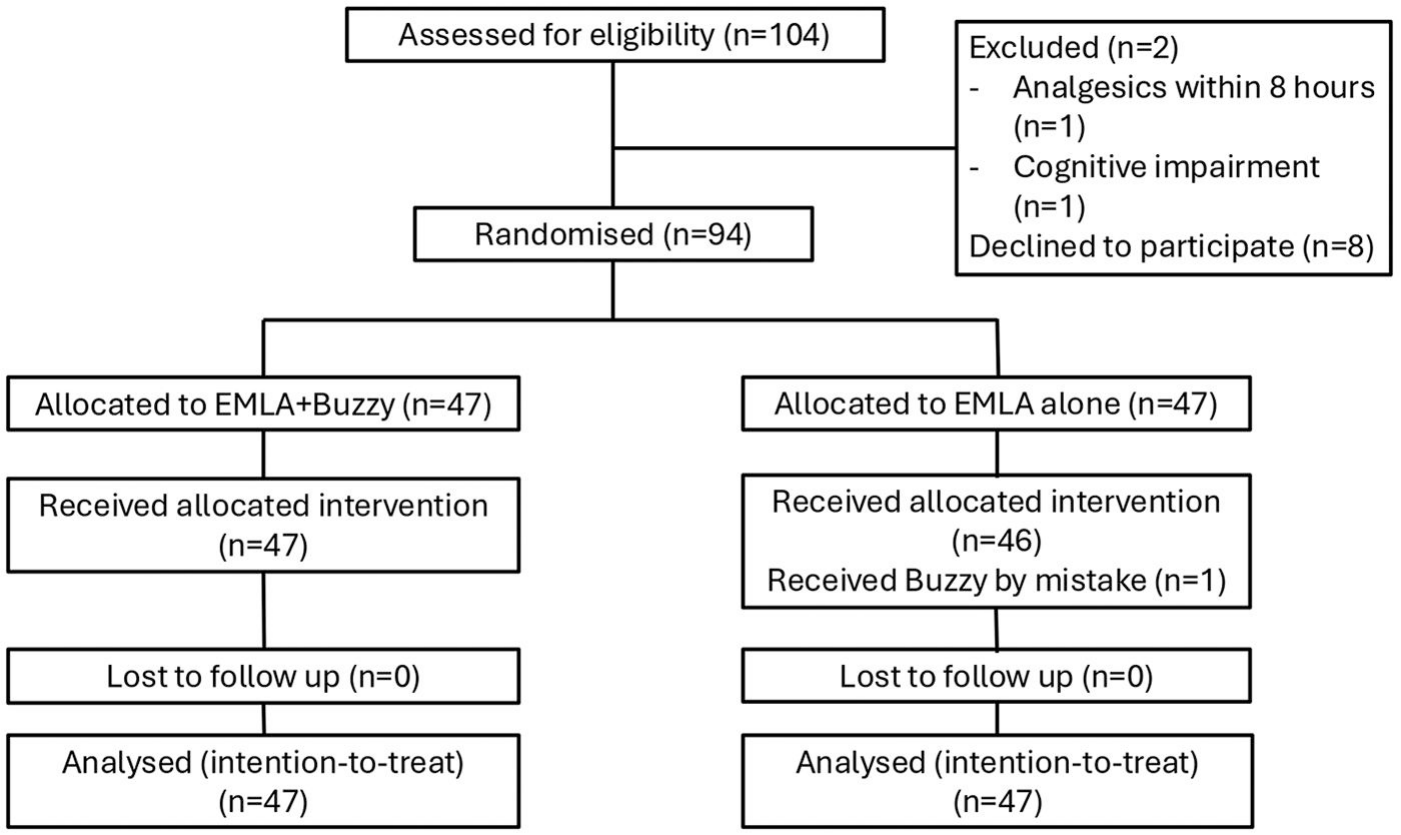
CONSORT flow diagram of participant enrolment, randomization, allocation, follow-up, and analysis (intention-to-treat).

Consequently, 47 children were analyzed in the EMLA plus Buzzy group and 47 in the EMLA alone group (Figure 1).

Baseline characteristics were comparable between the two groups (Table 1). The distribution of continuous variables was assessed using the Shapiro–Wilk test. As most variables showed a non-normal distribution (*p* ≤ 0.05), non-parametric tests were used for subsequent analyses. The median age was 8 years in both groups (IQR: 7–11 years in the EMLA plus Buzzy group; IQR: 6–11 years in the EMLA alone group). Procedures were performed mainly in the cubital fossa (88.3%).

**Table 1:** Patient characteristics continuous variables are presented as median (interquartile range) and compared using the Mann–Whitney test. Categorical variables are presented as number (percentage) and compared using the Chi-square or Fisher's exact test, as appropriate.

Patient characteristics	EMLA plus Buzzy Group (n = 47)	EMLA alone Group (n = 47)	*p* value
Age in years, median (IQR)	8.0 (7.0–11.0)	8.0 (6.0–11.0)	0.25
Sex, number (%)			0.15
Female	30 (63.8)	23 (48.9)	
Weight (kg), median (IQR)	29.0 (24.0–35.7)	26.0 (19.5–36.0)	0.18
Chronic disease, number (%)	2 (4.3)	6 (12.8)	0.27*
Site of the procedure, number (%)			0.52*
Cubital fossa	43 (91.5)	40 (85.1)	
Hand	4 (8.5)	7 (14.9)	

*Fisher's exact test.

Parent-assessed procedural distress, the primary outcome, was significantly lower in the EMLA plus Buzzy group, with a median CEMS score of 6 (IQR 5–7), compared with 9.5 (IQR 7–12) in the EMLA alone group (*p* = 0.001) (Table 2).

**Table 2:** Main study outcomes continuous variables are presented as median (interquartile range) and compared using the Mann–Whitney test. Categorical variables are presented as number (percentage) and compared using the Chi-square or Fishe*r's exact test, as appropriate.*

Procedure characteristics	EMLA plus Buzzy Group (n = 47)	EMLA alone Group (n = 47)	p value
CEMS—median (IQR)			
Parent	6.0 (5.0–7.0)	9.5 (7.0–12.0)	0.001
Healthcare Operator	6.0 (5.0–10.0)	7.0 (6.0–11.0)	0.07
FPS-R—median (IQR)			
Children	1.0 (0.0–2.0)	2.0 (0.0–4.0)	0.46
Parent	2.0 (0.0–2.0)	2.0 (0.0–4.0)	0.22
Healthcare Operator	0.0 (0.0–2.0)	1.0 (0.0–2.0)	0.82
Procedure successful, number (%)			
First attempt	42 (89.4)	40 (85.1)	0.76
Second attempt	4 (8.5)	7 (14.9)	0.52*
Third attempt	1 (2.1)	0 (0.0)	
Adverse events, number (%)			
Local burning with Buzzy use	3 (6.4)	0 (0.0)	
Child scared of Buzzy	1 (2.1)	0 (0.0)	
EMLA vasoconstriction	5 (10.6)	7 (14.9)	

*Fisher's exact test.

No relevant differences in pain intensity were observed between the two groups. Median children's self-reported pain was 1 (IQR 0–2) in the EMLA plus Buzzy group and 2 (IQR 0–4) in the EMLA alone group (*p* = 0.46). Likewise, pain scores reported by parents (*p* = 0.22) and healthcare operators (*p* = 0.82) were similar across groups (Table 2).

The first-attempt success rate was comparable, being 89.4% in the EMLA plus Buzzy group and 85.1% in the EMLA alone group (*p* = 0.76; Table 2).

Adverse events were reported in 15 patients (16.0%), mostly mild and mainly consisting of EMLA-related vasoconstriction. One patient experienced two adverse events, while the remaining 14 patients experienced one adverse event each. Three children reported experiencing local burning or fear related to Buzzy. No severe adverse event was recorded (Table 2).

To explore the potential influence of age on the study outcomes, a stratified analysis was performed (Table 3). In children aged ≤ 8 years, parent-reported distress was significantly lower in the EMLA plus Buzzy group compared with the EMLA alone group (median CEMS 6 vs. 9; *p* = 0.002). In contrast, no statistically significant differences were observed in children aged > 8 years. No relevant differences in pain scores or procedural success were found in either age group.

**Table 3:** Main study outcomes—stratified analysis continuous variables are presented as median (interquartile range) and compared using the Mann–Whitney test. Categorical variables are presented as number (percentage) and compared using the Chi-square or Fishe*r's exact test, as appropriate.*

	Age ≤8		
Procedure characteristics	EMLA plus Buzzy Group (n = 24)	EMLA alone Group (n = 25)	*p* value
CEMS—median (IQR)			
Parent	6.0 (5.0–7.0)	9 (7.0–12.0)	0.002
Healthcare Operator	6.0 (5.0–7.0)	8.0 (6.0–11.0)	0.05
FPS-R—median (IQR)			
Children	1.0 (0.0–2.0)	2.0 (0.0–4.0)	0.47
Parent	2.0 (0.0–2.0)	2.0 (0.0–4.0)	0.72
Healthcare Operator	0.0 (0.0–2.0)	1.0 (0.0–2.0)	0.48
Procedure successful, number (%)			
First attempt	22 (91.6)	20 (80.0)	0.42*
Second attempt	1 (4.2)	5 (20.0)	0.19*
Third attempt	1 (4.2)	0 (0.0)	

	Age >8		
Procedure characteristics	EMLA plus Buzzy Group (n = 23)	EMLA alone Group (n = 22)	*p* value
CEMS—median (IQR)			
Parent	6.0 (5.0–10.0)	10 (5.0–14.0)	0.10
Healthcare Operator	7.0 (5.0–10.0)	7.0 (6.0–11.0)	0.50
FPS-R—median (IQR)			
Children	2.0 (0.0–4.0)	2.0 (0.0–4.0)	0.79
Parent	1.0 (0.0–2.0)	2.0 (0.0–4.0)	0.18
Healthcare Operator	0.0 (0.0–2.0)	0.0 (0.0–2.0)	0.70
Procedure successful, number (%)			
First attempt	20 (87.0)	20 (90.9)	1.00*
Second attempt	3 (13.0)	2 (9.1)	

*Fisher's exact test.

## Discussion

This study showed that the combined use of EMLA cream and Buzzy significantly reduced procedural distress as perceived by parents during peripheral intravenous cannulation in children, compared with EMLA cream alone. A comparable pattern was observed in distress ratings reported by healthcare professionals, although the difference between groups did not reach statistical significance. In contrast, pain scores reported by children, parents, and healthcare professionals were low in both groups, with no statistically significant differences. In this context, the additional effect observed with Buzzy appears to be more closely related to the modulation of procedural distress than to further reductions in nociceptive pain when effective topical analgesia is already provided. In a stratified analysis, the reduction in distress appeared more evident in younger children (≤8 years), whereas no statistically significant differences were observed in older children. This finding should be interpreted with caution, given the limited sample size and the exploratory nature of the analysis. This is relevant in clinical practice, as distress experienced during needle procedures may influence children's response to subsequent procedures. Negative experiences can lead to increased anxiety, reduced cooperation, and the development of needle-related fear over time (2, 3).

This finding is plausibly related to the different mechanisms of action of the two interventions. EMLA cream reduces nociceptive input through peripheral sodium channel blockade, while Buzzy provides thermomechanical sensory stimulation, commonly explained by the gate control theory of pain, together with a sensory distraction component. According to this model, non-nociceptive sensory input can inhibit the transmission of nociceptive signals at the spinal level (11). In addition, central processes such as attention and sensory integration may also play a role in modulating both pain perception and behavioral responses (12). The complementary nature of these mechanisms may help explain the greater effect observed on distress compared with pain intensity when the two strategies are used in combination, as also suggested by recent randomized studies showing a synergistic effect of cold and vibration during venipuncture (20).

EMLA cream has been shown to reduce pain during venipuncture and intravenous cannulation in children (4, 6, 7), but its effect mainly targets nociceptive pain and does not address the emotional and behavioral components of procedural distress. Although its application may produce mild local vasoconstriction, previous studies have shown that it does not interfere with first-attempt success rate (7). Likewise, several randomized trials, as well as more recent systematic reviews and meta-analyses, have demonstrated that Buzzy is effective in reducing procedural pain and distress during venous cannulation without affecting technical success (10, 15, 16, 21), including in children with cognitive impairments (9).

In our study, median pain scores were low in both groups: 1.0 (IQR 0–2) in the EMLA plus Buzzy group and 2.0 (IQR 0–4) in the EMLA alone group. Pain scores in the combined group appeared slightly lower (by about 1–2 points) than those reported in studies evaluating Buzzy alone, without the use of topical anesthetics (10, 15).Lower anxiety and distress with Buzzy may help improve cooperation during the procedure. Discomfort was mild and transient, and no clinically relevant adverse effects were observed. Its use was acceptable to both children and caregivers. At the same time, Buzzy may be useful when a rapid approach is required, or topical agents cannot be used, particularly in time-constrained settings such as the emergency department (22).

The lower distress observed in the EMLA plus Buzzy group suggests that the emotional component of the procedure should be actively addressed. Although clinically important, the emotional and behavioral components of procedural distress are often overlooked in everyday practice, partly because of time constraints, limited training, and the lack of standardized protocols (5). Whether the effect observed in our study is specific to Buzzy or related to distraction mechanisms in general remains to be determined.

When comparing Buzzy with other non-pharmacological strategies, there is currently no clear evidence that one technique is consistently superior to another, as also highlighted in recent reviews (23). This is likely because different interventions act on distinct components of the procedural experience (24).

Distraction-based techniques mainly influence anxiety, distress, and the subjective perception of pain. For example, in some studies, including more recent randomized trials, Buzzy performed better than simple tools such as distraction cards during venipuncture, while showing comparable results to more engaging approaches such as audiovisual or interactive devices (8, 23). In addition, Buzzy has been shown to be more effective than simple techniques such as whistling in reducing both pain and fear during blood sampling (24).

Other distraction strategies have also been evaluated. Medical clowning has been shown to reduce both pain and anxiety during venipuncture, although its effect appears to be more pronounced on the emotional component rather than on nociceptive pain (25–27). To our knowledge, no studies have directly compared clown therapy with Buzzy. However, indirect evidence suggests that clown-based interventions may be less effective than more immersive approaches such as virtual reality in reducing procedural anxiety (28).

Music-based interventions represent another option. Both interactive music and active music production have been associated with reductions in pain and distress during venipuncture (29, 30). As with clown therapy, no direct comparisons with Buzzy are currently available.

Because Buzzy does not require active participation, it may be particularly suitable for younger children or for those with limited cooperation, including children with cognitive impairment (9, 25). Its effect is not uniform and seems to vary across settings and procedures, as well as according to patient characteristics such as baseline anxiety.

First-attempt cannulation rates were comparable between groups, indicating that the use of Buzzy did not affect procedural success. Similar findings have been reported in previous and more recent studies, in which vibration and cold stimulation were not associated with difficulties in venous cannulation (9, 10, 31).

Taken together, these findings are consistent with a multimodal, child-centered approach to venous cannulation in pediatrics, in which topical anesthesia addresses nociceptive pain while complementary distraction-based strategies are used to minimize procedural distress.

### Clinical implications

Adding Buzzy to topical anesthesia may help reduce procedural distress during peripheral intravenous cannulation in children, without affecting technical success. This can be relevant in routine clinical practice, where distress and cooperation often influence how easily the procedure is performed. Given its ease of use and good tolerability, Buzzy may represent a practical adjunct to topical anesthetics, especially in children with higher levels of anxiety or previous negative experiences.

### Study limitations

This study presents some limitations. First, it was not blinded, because blinding of participants, healthcare professionals, and evaluators was not possible.

Secondly, distress was assessed by parents and healthcare operators, but not directly by the children. However, commonly used distress rating scales are observational scales, and we did not find suitable tools for self-reporting distress in children aged 4–12 years.

Thirdly, although a stratified analysis by age was performed, the study was not powered to detect differences between age groups, and these findings should therefore be interpreted with caution. In addition, no stratified analysis by sex was conducted.

Potential confounding factors, such as parental behaviour during the procedure and differences in healthcare professionals' interaction, were not systematically recorded and could not be adjusted for in the analysis, which may have influenced the observed levels of distress.

In addition, most procedures were carried out by experienced nursing staff working in tertiary-level pediatric hospitals, and the findings should be interpreted within this clinical context.

A further limitation concerns the assessment tools. Although CEMS has been validated in children aged 7–12 years, we also applied it to younger children because it is based on observable behavioral manifestations rather than self-report. This should be considered when interpreting the study findings.

Finally, the study did not include a comparison arm with EMLA plus a standard non-pharmacological distraction technique, which would have allowed us to determine whether the effect on distress was specific to Buzzy or related to distraction in general.

## Conclusion

The combination of EMLA cream and Buzzy for peripheral intravenous cannulation significantly reduced procedural distress, according to parents' perception, without significantly affecting pain scores. These findings suggest that addressing distress, in addition to pain, may be clinically meaningful in pediatric venous cannulation.

Future studies directly comparing Buzzy with other validated distraction strategies, in combination with topical anesthesia, may help clarify whether its effect is unique or part of a broader attentional modulation phenomenon.

## Data Availability

The raw data supporting the conclusions of this article will be made available by the authors, without undue reservation.
